# Equalisation of alcohol participation among socioeconomic groups over time: an analysis based on the total differential approach and longitudinal data from Sweden

**DOI:** 10.1186/1475-9276-10-10

**Published:** 2011-02-10

**Authors:** Jean-Baptiste Combes, Ulf-Göran Gerdtham, Johan Jarl

**Affiliations:** 1 Health Economics Research Unit (HERU), University of Aberdeen, Polwarth Building, Foresterhill, Aberdeen AB25 2ZD, UK; 2 National School for Statistics and Information Analysis (ENSAI), Campus de Ker-Lann, Rue Blaise Pascal, BP 37203, 35172 Bruz Cedex, France; 3 Health Economics & Management, Institute of Economic Research, Lund University, P.O. Box 117, SE-221 00 Lund, Sweden; 4 Center for Primary Health Care Research, Malmö University Hospital, Lund University/Region Skåne, SE-205 02 Malmö, Sweden; 5 Economics Department, Lund University, P.O. Box 117, SE-221 00 Lund, Sweden

## Abstract

**Background:**

Health inequality and its social determinants are well-studied, but the determinants of inequality of alcohol consumption are less well-investigated.

**Methods:**

The total differential approach of decomposition of changes in the concentration index of the probability of participation in alcohol consumption was applied to 8-year longitudinal data for Swedish women aged 28-76 in 1988/89.

**Results:**

Alcohol consumption showed a pro-rich inequality, with income being a strong contributor. Overall participation remained fairly constant, but the inequality decreased over time as abstinence became less common among the poor and more common among the rich. This was mainly due to changes in the relative weights of certain population groups, such as a decrease in the proportional size of the oldest cohorts.

**Conclusions:**

Inequality in participation in alcohol consumption is pro-rich in Sweden. This inequality has tended to decrease over time, due to changes in population composition rather than to policy intervention.

## Background

The statistical association between socioeconomic circumstances (income, wealth, socioeconomic status, occupational group, wealth, or educational level) and morbidity/mortality, or their positive dual indicators health status/life expectancy, is well documented in a solid body of academic research covering many years and many countries.

The bulk of recent health economic research on socioeconomic health inequality has been undertaken within the seminal ECuity Group, which has been in the forefront of developing analytical methods for measurement and explanation of socioeconomic health inequality [1].

In a recent article, Van Doorslaer & Koolman [2] found significant income-related health inequality in 13 European Union (EU) member states (excluding Sweden), using the concentration index and decomposition techniques. The inequality was particularly high in Portugal, and fairly high in the UK and Denmark. Relatively low health inequality was observed in The Netherlands and Germany, but also in Italy, Belgium, Spain, Austria, and Ireland. Moreover, the authors found a positive correlation with income inequality, but concluded that health inequality is not merely a reflection of income inequality. In a decomposition analysis, they showed that in terms of explaining cross-country differences in income-related health inequality, the elasticities of the explanatory variables are generally more important than their unequal distribution by income. This raises the question of whether inequality in the determinants of health causes inequality in health.

One dimension of health determinants is health-related behaviours [3,4], including several factors such as smoking, excessive nutritional intake, and alcohol consumption. The impact of lifestyles on health inequality is a current debate across disciplines. A recent survey indicates that there has been an underestimation of the impact of health-related behaviours on health inequality, and that the impact of socioeconomic characteristics on health inequality can be attenuated by the introduction of health-related behaviours [5]. The study presented in this article focuses on alcohol consumption, which causes adverse effects both for individuals and for society, for example by increasing the risk of several diseases, reducing productivity, and increasing costs in the criminal justice system [6]. Alcohol consumption is thus considered a public health issue [7]. It is an ambiguous health determinant, as it has been shown to have protective effects on an individual level, mainly in terms of reducing the risk of certain diseases [6]. The net effect, however, is considered detrimental on average, even for low consumption [8]. Low alcohol consumption is also associated with a number of positive outcomes with regard to the labour market, such as increased pay and reduced sickness absenteeism. Thus, although the ambiguity of the effects makes alcohol consumption a difficult health determinant to study, as the negative effects of consumption are substantial in western countries today, it is considered worth the effort.

The increase in alcohol consumption in Sweden during recent years (about a 25% increase 1989 - 2006 [9]) is expected to increase both individual and societal harm. The harm increase is likely to be larger in Sweden compared to other European countries, as the Swedish pattern of consumption, weekend binging, is one of the most harmful consumption patterns [7,10]. However, it is unlikely that the societal harm of alcohol consumption and its expected rise will be evenly distributed in society. Alcohol consumption differs greatly on an individual level, and probably also differs between population groups, with some groups even having the potential to benefit in terms of health. Understanding which groups suffer (the most) negatively from alcohol consumption is important, in order to design and implement effective public health interventions to increase individual and societal health status. A first step in this direction is to determine which socioeconomic groups are more likely to consume alcohol. Once this is established, it is important to look at the characteristics of those groups. Studying inequalities in alcohol consumption is one way of doing this.

To our knowledge, there are currently no models that bring together inequalities in health behaviours and health. Nevertheless, some empirical studies have investigated the relationship between health-related behaviours and income or social hierarchy. A Singaporean study by Fong *et al. *[11] showed that less-educated people are more likely to have unhealthy behaviours such as smoking, drinking alcohol, and not doing any exercise, while Borrell *et al. *[12] showed in a Spanish setting that unhealthy behaviours are differentially distributed among social classes. In the case of tobacco consumption, this distribution changes over time, with the risky health behaviour becoming more concentrated among the lower social classes [13].

Regarding alcohol consumption, Casswell *et al. *[14] showed that people with higher incomes drink more often (both men and women), although educational and occupational activities do not appear to have a strong impact on frequency. It is also interesting to note that better-educated people consume smaller amounts of alcohol [14]. Lantz *et al. *[15] showed that risky behaviours are more prevalent among lower income levels, and that lower income leads to an increase in mortality risk. At odds with this are the results from the English Health Survey 2006, showing that higher income groups consumed alcohol more frequently and binged more often compared to lower income groups [16]. A Spanish study in turn reported that men and women of upper social class (compared to lower social class) are proportionally more represented among light alcohol consumers, while at the same time upper class women (compared to lower social class) are also more represented among excessive consumers and less among non-drinkers. There are no significant differences between social classes for men regarding excessive consumption [12]. In the UK, more heavy male drinkers are found among manual workers, while for women it is always the upper classes that drink more [17].

Regarding the debate over whether social status or health-related behaviours have the greatest impact on self-assessed health, Contoyannis and Jones [3] showed that lifestyle accounts for 12% of the effect of social status on health and 27% of the effect of education on health. Similarly, Balia and Jones [4] showed that lifestyle contributes strongly to the inequality in mortality, reducing the effect of socioeconomic status.

Evidence from prior research indicates that health-related behaviours are unevenly distributed across the population, based on different indicators such as social class, education, and income. However, there seem to be large differences between different behaviours as well as between genders, and some of the above mentioned studies suggest that the Grossman model from Dardanoni & Wagstaff [18] does not hold for alcohol, as the model predicts that the rich should engage less in detrimental health behaviours. It is therefore of interest to thoroughly identify the socioeconomic groups participating in the consumption of alcohol. Moreover, it seems important to go one step further and evaluate the characteristics of the groups participating in alcohol consumption.

### Aim of the study

The aim of the present study was to investigate income-related inequalities in alcohol consumption, a determinant of inequalities in health, and its changes over an eight-year period. The specific research questions were:

1. Are inequalities in income-related alcohol participation more concentrated among the rich or the poor?

2. What characteristics explain this inequality at different time points?

3. What is the pattern of inequality over time and how can it be explained?

These research questions were studied in a longitudinal setting using concentration index methods and decomposition techniques, in particular the total differential approach. Using the total differential approach in combination with longitudinal data, we aimed to explain changes in inequalities over time by analysing the driving forces of the inequality. A special feature of the study is that it focuses on inequalities in alcohol participation over the long term in an ageing cohort. We followed a random population sample of Swedish women from 1988/89 over an eight-year period. The effects of alcohol consumption were expected to differ between genders [19]. Due to space limitations, we chose to present the results for women in full, and briefly discuss the results for men. Initial inspection of the data confirmed the existence of gender differences.

The results of our empirical analyses led us to the following conclusions. Alcohol consumers are more concentrated among the rich. Income and having children contribute to a pro-rich inequality, while individual heterogeneity (IH), that is, characteristics of individuals that do not change over time and that are not measured, contributes to pro-poor inequality. This inequality in participation decreases over the study period, but not statistically significantly. Since the prevalence of participation in alcohol consumption was approximately constant over this period, the decrease in inequality was driven by abstention becoming less common among the poor and more common among the rich. The decomposition of the decrease in inequality in participation showed that this decrease was mainly due to changes in population patterns, and was counterbalanced by unexplained factors; for example, the decrease of the oldest cohorts (ageing effect) was a factor in the decrease of the inequality towards a more equal drinking participation.

This article proceeds as follows; the data material is described in the next section, which is followed by a method section where we discuss the concentration index and decomposition. After this follow the results, and finally we discuss the results and provide the conclusions of the study.

#### Data material

The study data were drawn from the Survey of Living Conditions (the ULF survey) and linked to income data from the National Income Tax Statistics [20,21]. Each year since 1975, Statistics Sweden has conducted a systematic survey of living conditions in the form of 1-hour personal interviews with randomly selected adults aged 16-84 years. In the current study, we used data from individuals who were included in the 1980/81 wave (W1) and who also responded in the 1988/89 wave (W2) to analyse the effect of ageing on socioeconomic inequality in alcohol consumption between W2 and the 1996/97 wave (W3). Data on alcohol consumption were available only for W2 and W3.

W1 and W2 had 5,106 individuals in common, while 3,780 individuals responded to all three waves. We excluded individuals younger than 20 years or older than 68 years at the baseline year W1, to allow an ageing effect over the three waves. The final sample thus included 2,115 women (2,048 men) in W1 and W2 and 1,796 women (1,623 men) in W3. The variable for income had 39 missing observations in W1, 33 in W2, and 27 in W3 (all recoded; see below). Other variables had few missing values, so we recoded them into the most prevalent group. Attrition between W2 and W3 was not taken into account. A sensitivity analysis showed that there was very little difference between the two populations, except in terms of age, with dropouts being more likely to be old.

### Alcohol

Several questions about alcohol consumption were included in the survey. We use a question that was asked both in W2 and in W3, namely whether the respondent had consumed any alcoholic beverage in the last 12 months, to construct a binary variable for participation (equal to 1 if the respondent had consumed alcohol, 0 otherwise); see Table 1. We also construct an intensity variable in the form of a continuous variable measuring how many grams of pure alcohol the respondent consumed. This variable is based on a battery of questions regarding the amounts of different alcoholic beverage consumed by the respondent during a week, and a conversion of the answers into grams of pure alcohol. We only use this variable in the sensitivity analysis, as the questions were slightly differently phrased between waves, raising concerns that the variable might not be perfectly comparable between waves.

**Table 1:** Alcohol participation

N (%)	1988/89		1996/97	
	Women	Men	Women	Men
Participation in alcohol consumption	1,694 (80.1%)	1,807 (88.2%)	1,435 (79.9%)	1,431 (88.2%)

### Full income

The full income variable consists of two components, annual disposable income and annuity of net wealth. The dataset contains information about disposable income net of taxes (income from capital, employment and business, and all income transfers). Information regarding taxable net wealth was taken from National Income Tax Statistics in Sweden and converted to net wealth at market value following the method described in Gerdtham & Johannesson [22]. Annuity of net wealth is based on life expectancy in Sweden, differentiated for gender and age, and a 3% interest rate[23]. This measure also includes value of property, a variable that had a few missing values for W3 (n = 99; 0.84% of the sample). These missing values were replaced by the median property value, adjusted for age, gender, and cohort. Imputation of missing values has a tendency to reduce the standard errors of any statistics involving income; however, imputation involved only 99 observations and did not change the standard error of the variable by much (70,641.5 without to 70,633.2 with imputing, while the mean changed from 92,471.8 to 92,514.6). Both income measures were converted into 1997 prices using the consumer price index, and added together to obtain full income. In order to transform the household income into individual (adult) income, considering that the needs of a household do not increase proportionally with added members, we applied the OECD-modified equivalence scale. This scale, first proposed by Haagenars et al. [24], assigns a value of 1 for the first adult, 0.5 for each additional adult, and 0.3 for each child. The regression analysis includes both current income and long-term (mean) income over the study period (1988/89-1996/97).

### Health

Health was measured with a self-reported health question on general health, with categories of bad, moderate, and good health. We use health at W1 in the estimations, in order to reduce any potential reversed causality of alcohol consumption on health. Self-assessed health is considered to have a good prediction of mortality and morbidity [25], and captures the general health aspect relevant for this study.

### Other variables

Age was divided into six groups (<35 years old, 35< = age < 45, 45< = age < 55, 55< = age < 65, 65< = age < 75, age > = 75), and we also used a cohort variable (born after 1947, 1932< = cohort < 1947, 1924< = cohort < 1932, 1916< = cohort < 1924, cohort < 1916). The cohort variable is constructed to represent different number of years in each cohort with large number of years in the younger cohort. Therefore it does not match with the age category variable. This will help avoid multi collinearity and still be able to measure the age and cohort effects. A dummy variable was also included for the period effect. Following Portrait *et al. *[26] who explain the different methods of handling the collinearity problem caused by controlling for cohort, age and period, dummies are used in the current study. Socioeconomic status is described by an educational and an occupational variable. Four educational groups are used: no education or primary school level, vocational high school studies, academic high school level, and university level. Occupational status is described by a six-outcome variable: employed, self-employed, student, unemployed, retired, and homemaker. Parental socioeconomic status is categorised into four groups for each parent separately: white-collar, self-employed, working class, and missing data. The group variable for missing data is included because 5% of the population did not answer the question for the father in W2, 3.5% did not answer for the father in W3 (Table 2), and 62% did not answer for the mother. This category thus corresponds to non-response in the data set. However, it does not tell us if it is a random or non-random non-response, e.g. if the person grew up in a single-parent family. Marital status is included as a dummy variable. The number of children in the household is included as three dummy variables (1 child, 2 children, and 3 or more children, with a baseline of no children). Table 2 gives the means for women of the independent variables.

**Table 2:** Means and concentration indexes (Cs) of independent variables

Wave	1988/89	1996/97		1988/89	1996/97	
**Variables**:	**Mean**:	**Mean**:	**Diff**:	**Cs**:	**Cs**:	**Diff**:
**Age**						
20< = age < 35	0.1486	0	-14.86%	-0.2041	0.0000	20.41%
35< = age < 45	0.2438	0.2037	-4.01%	-0.0247	-0.1993	-17.46%
45< = age < 55	0.1857	0.2694	8.36%	0.1417	0.0276	-11.41%
55< = age < 65	0.1810	0.1852	0.42%	0.0280	0.1384	11.04%
65< = age < 75	0.1948	0.1807	-1.41%	0.0068	0.0038	-0.30%
75< = age < = 84	0.0461	0.1611	11.49%	0.0875	0.0454	-4.21%
**Cohort**						
Cohort > = 1947	0.3276	0.3541	2.65%	-0.1277	-0.1251	0.26%
1932< = cohort < 1947	0.2962	0.3137	1.75%	0.1140	0.1177	0.37%
1924< = cohort < 1932	0.1429	0.1425	-0.03%	0.0431	0.0318	-1.12%
1916< = cohort < 1924	0.1590	0.1352	-2.38%	-0.0218	-0.0078	1.40%
Cohort < 1916	0.0743	0.0544	-1.99%	0.0768	0.0790	0.22%
**Father's socioeconomic status**						
White-collar	0.1852	0.1970	1.17%	0.1610	0.1706	0.95%
Working class	0.4314	0.4349	0.35%	-0.0722	-0.0763	-0.41%
Self-employed	0.1461	0.1487	0.25%	0.0541	0.0383	-1.58%
Farmer	0.1862	0.1841	-0.21%	-0.0552	-0.0506	0.45%
Missing	0.0510	0.0354	-1.56%	0.0665	0.0978	3.14%
**Mother's socioeconomic status**						
White-collar	0.0814	0.0881	0.67%	0.0357	0.0360	0.03%
Working class	0.1800	0.1869	0.69%	-0.0856	-0.0685	1.71%
Self-employed	0.0395	0.0382	-0.14%	0.0157	0.0414	2.57%
Farmer	0.0714	0.0713	-0.02%	-0.1149	-0.0852	2.97%
Missing	0.6276	0.6156	-1.20%	0.0321	0.0230	-0.90%
**Immigrant**						
First-generation immigrant	0.0752	0.0730	-0.23%	-0.0148	-0.0131	0.17%
**Marital Status**						
Single	0.2752	0.3215	4.63%	0.3996	0.3309	-6.88%
**Children**						
No children	0.6157	0.7800	16.43%	0.0797	0.0795	-0.02%
One child	0.1438	0.0965	-4.73%	0.0143	-0.1923	-20.67%
Two children	0.1610	0.0898	-7.12%	-0.1292	-0.2868	-15.76%
Three or more children	0.0795	0.0337	-4.59%	-0.3844	-0.5155	-13.11%
**Education**						
No education, or primary school only	0.3929	0.3608	-3.20%	-0.1035	-0.0835	2.00%
Vocational high school studies	0.3343	0.3457	1.14%	-0.0530	-0.0716	-1.86%
Academic high school level	0.0548	0.0516	-0.31%	0.0860	-0.0036	-8.95%
University level	0.2181	0.2419	2.38%	0.2438	0.2278	-1.60%
**Income**						
Income	11.3028	11.3917	8.89%	0.0173	0.0232	0.59%
**Occupation**						
Employed	0.6024	0.4983	-10.41%	0.0671	0.0345	-3.26%
Self-employed	0.0329	0.0028	-3.01%	-0.2625	-0.5860	-32.34%
Student	0.0071	0.0084	0.13%	0.0351	-0.4030	-43.81%
Unemployed	0.0148	0.0326	1.78%	-0.1475	-0.1817	-3.42%
Retired	0.2890	0.4024	11.33%	-0.0043	0.0133	1.76%
Homemaker	0.0519	0.0544	0.25%	-0.5213	-0.2177	30.37%
**Health**						
Good health	0.7724	0.7980	2.56%	0.0128	0.0130	0.01%
Moderate health	0.1876	0.1694	-1.81%	-0.0349	-0.0523	-1.73%
Bad health	0.0400	0.0325	-0.75%	-0.0831	-0.0444	3.88%

Reading table 2: the proportion of people reporting themselves as single increased by 4.63% between the two waves, from 27.5% to 32.1%. The income-related concentration index is pro-rich, meaning that being single is a characteristic more concentrated among the rich; it decreases by 6.9% over the years, i.e. being single is a characteristic that is less concentrated among the rich in the second wave.

## Methods

### Measurement of inequalities

Inequalities are widely measured by the concentration curve and the concentration index (C), both of which are related to the Lorenz curve and the Gini coefficient method. We preferred the use of C instead of other dispersion measure such as the range ratio, as we were interested in the decomposition property of C (see below). The concentration curve is a plot of the individuals, ranked by a socioeconomic variable from the lowest to the highest rank with a cumulative alcohol consumption variable distribution. For a complete description, see the World Bank report [27]. We calculate C to measure income-related inequalities in alcohol consumption using the covariance formula:

(1) $$C=\frac{\text{2}}{\mu }\text{cov(}y_{i},R_{i})$$

where *μ *is the mean of the participation in alcohol consumption, *y_i _*is the participation variable, and *R_i _*is the rank of people by income. This formula makes it clear that C is just a modified covariance between the socioeconomic rank and the alcohol consumption variable. For a complete description of the evolution of the Gini coefficient, from which C has branched off, see Xu [28] and the World Bank report [27]. C can be computed on both continuous and binary variables. For a binary variable, Wagstaff [29] proposes normalisation of C, by dividing it by one of the bounds of C. These bounds are 1-*μ *and 1+*μ*, where *μ *is the mean of the variable, which in the case of a binary variable is a proportion [29]. We normalise C using the lower bound.

We use two income ranking variables; one is the current income, and one is a measure of long-term (mean) income over the waves (1988/89-1996/97) for the same individual. We focus on the latter income measure in the analysis, in order to prevent a re-ranking effect that could bias our interpretations.

### Decomposing inequalities

We decompose C in order to analyse which factors impact the C of alcohol participation. The decomposition is based on the formulas given in Wagstaff *et al. *[30]:

(2) $$C=\frac{\text{2}}{n\mu }\sum_{i=\text{1}}^{n}R_{i}y_{i}-\text{1}$$

and uses a linear regression model:

(3) $$y_{i}=\alpha +\sum_{k}\beta_{k}x_{ki}+\varepsilon_{i}$$

where *y_i_*, *μ *and *R_i _*are defined as above, *x_ki _*are a set of k regressor variables for the i individuals, and *ε_i _*is the error term. By substituting (3) in (2), we obtain:

(4) $$C=\sum_{k}\frac{\beta_{k}{\bar{x}}_{k}}{\mu }C_{k}+\frac{GC_{\varepsilon }}{\mu }$$

where ${\bar{x}}_{k}$ is the mean of *x_k _*(the characteristic k) and *C_k _*is the concentration index for *x_k_*, defined analogously to C. $\frac{\beta_{k}{\bar{x}}_{k}}{\mu }$ is the elasticity of the alcohol consumption variable with respects to the explaining variable *x_k_*. The last term, $\frac{GC_{\varepsilon }}{\mu }$, can be computed as a residual. The decomposition result in (4) relies on the fact that the alcohol consumption variable is additive in its component x.

Here, we are performing a decomposition analysis on a binary variable using a probit random effect model. Probit models are based on unobservable latent variables; the decomposition is performed on this latent variable and not on the observed categorical measure of health. Consequently, it is only the explained variation in the health measure that can be decomposed [31]; the error term is equal to 0 and the percentage of explained inequalities cannot be computed.

To sum up, the decomposition gives two general terms for each of the characteristics we put in the model, and is based on a regression with the alcohol participation variable as the dependent variable. The first term is the elasticity of the variable to the participation in alcohol consumption. A positive elasticity means that the individuals with this characteristic are more likely to participate in alcohol consumption. The second term is the dispersion of the characteristics along the income distribution. If positive, the characteristic is more concentrated among the rich. The product of the two terms gives the total contribution of the characteristic. The interest of the analysis is that a strong contribution towards the alcohol participation C being more concentrated among the rich could be the result of a characteristic representing individuals with a negative elasticity (individuals are less likely to consume alcohol) and a negative income concentration (individuals with this characteristic are more concentrated among the poor). The two terms are negative, which means that the characteristic contributes to a alcohol participation C being more concentrated among the rich.

### Bootstrapping differences over waves

It is not possible to do a Student test to assess the statistical significance of the difference in C over the two waves, as the observations are not independent. Consequently, we resample the income and alcohol variables to compute 6 000 C and assess non-parametrically if there is a significant difference between the two waves.

### Total differential approach of the changes in inequality in alcohol consumption

The total differential approach (TDA) decomposes the change in the contributing factors of C; it is also based on the same regression as the first decomposition. In the first decomposition we had two contributing factors: the elasticity and the dispersion of the characteristic along the income distribution. We would like to know the overall impact and the impact of both terms in the change over time. Wagstaff *et al. *[30] describe two methods to decompose the changes over time: i) Oaxaca decomposition [32], where the changes in C are decomposed into the changes and elasticities of the determinants of health (alcohol consumption in this case), and ii) the TDA [30], where the changes in C are decomposed through the differentials in means and Cs of the determinants of alcohol consumption. One disadvantage of the well-known Oaxaca decomposition is that it is difficult to disentangle changes within the elasticity (whether it is the parameter estimation or the mean of the explaining variable), while the TDA allows for changes in each component of equation (3). On the other hand, the TDA is an approximation of the variations of C, and is accurate only for small changes. Consequently, we add a sensitivity analysis by running Oaxaca decomposition. Two formulas are available for the Oaxaca decomposition, and they are not perfectly equivalent:

(5) $$\Delta C=\sum_{k}\eta_{kt}(C_{kt}-C_{kt-\text{1}})+\sum_{k}C_{kt-\text{1}}(\eta_{kt}-\eta_{kt-\text{1}})+\Delta \frac{GC_{\varepsilon t}}{\mu_{t}}$$

(6) $$\Delta C=\sum_{k}\eta_{kt-\text{1}}(C_{kt}-C_{kt-\text{1}})+\sum_{k}C_{kt}(\eta_{kt}-\eta_{kt-\text{1}})+\Delta \frac{GC_{\varepsilon t}}{\mu_{t}}$$

Where *η_kt _*is the elasticity of the alcohol consumption variable. These formulas need to be adjusted for the binary variable case [33], but this adjustment still does not answer the critique that it is impossible to disentangle the effect within the elasticity. Consequently, Wagstaff [30] proposes an approximation to the difference by taking the total differential of equation (3). In our case (as explained below, the estimated parameters are constant over time) the result is:

(7) $$dC=\sum_{k}\frac{\delta C}{\delta {\bar{x}}_{k}}d{\bar{x}}_{k}+\sum_{k}\frac{\delta C}{\delta C_{k}}dC_{k}+d\frac{GC_{\varepsilon t}}{\mu }$$

(8) $$dC=\sum_{k}\frac{\beta_{k}}{\mu }(C_{k}-C)d{\bar{x}}_{k}+\sum_{k}\frac{\beta_{k}{\bar{x}}_{k}}{\mu }dC_{k}+d\frac{GC_{\varepsilon t}}{\mu }$$

Proofs are given in Podder [34] and Wagstaff [30]. The TDA takes the two contribution factors of the first decomposition (elasticity and dispersion of the characteristic) and calculates their contributions to the change. The only term within the elasticity that changes is the mean of the characteristic. The effect of a positive change of the mean could lead to different sign depending on some initial values (parameters, dispersion of the characteristic, dispersion of the participation in alcohol consumption). The same applies to a change in the concentration of the characteristic. However it has to be interpreted as the effect of a change of the mean or of the dispersion of the characteristic, as they are the only two terms that change. The sum of the two terms gives the total contribution to the change of the characteristic. The two terms could have major changes in opposite directions between the two time periods, which would reduce the contribution to nothing. No change in one of the two contributors to the C will lead to no contribution to the change of this contributor. In that case, all the characteristic's contribution to the change will come from the other contributor.

Explanation following the formulas (column numbers refer to the results as presented in Table Six):

Columns 1 and 3 correspond to $\sum_{k}\frac{\beta_{k}}{\mu }(C_{k}-C)d{\bar{x}}_{k}$ and $\sum_{k}\frac{\beta_{k}{\bar{x}}_{k}}{\mu }dC_{k}$ respectively from equation 8. Consequently, column 1 (column 3) gives the direct and indirect effects of ${\bar{x}}_{k}$ (*C_k _*respectively), which are given by the term $\frac{\beta_{k}}{\mu }(C_{k}-C)$ ($\frac{\beta_{k}{\bar{x}}_{k}}{\mu }$), and the effect of a change in ${\bar{x}}_{k}$ (*C_k_*), given by the term $d{\bar{x}}_{k}$ (*dC_k_*). Interpretations are quite straightforward because $\frac{\beta_{k}}{\mu }(C_{k}-C)d{\bar{x}}_{k}$ ($\frac{\beta_{k}{\bar{x}}_{k}}{\mu }dC_{k}$) is the exact effect of ${\bar{x}}_{k}$ (*C_k_*). However, an observed increase in ${\bar{x}}_{k}$ (*C_k_*) could lead to either a decrease or an increase in alcohol-related inequality, depending on the sign of $\frac{\beta_{k}}{\mu }(C_{k}-C)$ ($\frac{\beta_{k}{\bar{x}}_{k}}{\mu }$). Observing no variation, $d{\bar{x}}_{k}=\text{0}$ or *dC_k _*= 0 would have led to no effect, which implies that the actual C would have decreased (increased) if the figure observed in column 1 or column 3 is positive (negative). Consequently, the variation in ${\bar{x}}_{k}$ (*C_k_*) is the first cause of the effect, but the magnitude and sign also depends on $\frac{\beta_{k}}{\mu }(C_{k}-C)$ ($\frac{\beta_{k}{\bar{x}}_{k}}{\mu }$). Variations in ${\bar{x}}_{k}$ (*C_k_*) can be found in Table Two.

We note that as the TDA is an approximation, there is a supplementary error term caused by the fact that the TDA does not take into account all of the differences between the Cs (for a probit estimation we have to use the explained Cs instead of the "true" Cs). In short, we will have the "true" C (eq. 1), the explained C (eq. 4), and the explained change of the explained C (eq. 8).

### Panel Estimation

The decomposition analysis above requires an estimation of the determinants of alcohol participation. Panel regression analysis may be more accurate than a pooled analysis when the unobserved effect is correlated with the explanatory variables, since it allows us to control for the potential confounding factor of IH [35]. If the unobserved effect contains an individual random variable *k *that is correlated to the observed variables, then we cannot consistently estimate the parameters. We can take into account a constant characteristic of individuals over time using the panel structure [36]. Two main techniques have been used to consistently estimate the parameters in the presence of the IH: the random effect estimation (RE) and the fixed effect estimation (FE). The former constrains *k_i _*to be orthogonal to *x_it_*, and puts *k_i _*in the error term. These assumptions are stronger than those needed for a pooled regression, but in using generalised least squares (GLS) we account for the implied serial correlation in the composite error term *v_it _*= *k_i _*+ *u_it_*. While the correlation between the individual effect and the explanatory variable is neglected by the RE approach [37], the FE explicitly takes this correlation into account [36]. Thus, the FE specification eliminates the IH and consistently estimates the parameters, though it also eliminates all time-invariant variables except in the cases where they interact with time-varying variables. Mundlak [37] proposed the introduction of an auxiliary regression to decompose the heterogeneity effect into a correlation effect with the explanatory variables and a term that has no correlations with *x_it_*. In practice, we introduce the mean of *x_it _*in the regression analysis to account for the correlation between the IH and the explanatory variables, and we estimate the model with the GLS method used for RE [38,39]. However, we can apply this method only for time-varying variables; otherwise it will lead to collinearity. As a consequence, we assume that there is no correlation between the time-constant variables and IH, or alternatively that we cannot distinguish between the effects of IH and the time-constant variables. The Mundlak technique is also accurate for binary outcomes; its accuracy is described in Wooldridge [36] and was used by Diaz-Serrano [40] and Contoyannis *et al. *[41]. The variables for which we introduce the mean are marital status, having children, education, and socioeconomic status. We also introduce the mean of income but do not include it explicitly as a Mundlak technique variable, as we will be using a different interpretation (see the discussion section).

## Results

We first report Cs of participation in alcohol consumption for women in different waves (men are discussed briefly below). Our analyses are gender-specific because the effect of alcohol consumption differs between genders [19]. This is followed by the results from the binary probit analyses of the determinants of participation in alcohol consumption. Finally, we report estimations of contributions of the determinants of alcohol consumption to the Cs for each wave, and explain the changes in Cs across waves. The results section ends with extra analyses; that is, the results for men, the Oaxaca decomposition, and the results of an alternative alcohol variable measuring the level of alcohol consumption (intensity).

### Inequality in alcohol consumption based on current and mean income rankings

Tables 3 and 4 give the Cs for participation in alcohol consumption for both waves, based on long-term mean income and current income, respectively. The Cs are consistently positive, indicating that alcohol consumers are concentrated towards the better-off (i.e. there is a pro-rich alcohol participation inequality). While the *current *income-related inequality in participation in alcohol consumption increases over time (0.04836; p = 0.1625), the *long-term full *income-related inequality decreases numerically over time (-0.0232; p = 0.3105). Decomposing the changes over time is still of interest even though the change is not significant, because we will observe what characteristics are linked to a decrease or an increase in inequality.

**Table 3:** Concentration indexes for women (current income)

Current income	Consumers vs. Abstainers
	Women (Normalised value; p-value)
W2	0.1151 (<0.001)
W3	0.1670 (<0.001)
W3-W2*	0.0486 (p = 0.1625)

* Bootstrap difference

**Table 4:** Concentration indexes for women (long-term income)

Long-term income	Consumers vs. Abstainers
	Women (Normalised value; p-value)
W2	0.1485 (<0.001)
W3	0.1271 (<0.001)
W3-W2*	-0.0232 (p = 0.3105)

* Bootstrap difference

### Binary probit regression of the determinants of alcohol participation

Column 2 in Table 5 presents the parameter estimation of the regressions for participation in alcohol consumption. Women aged over 35 are more likely to consume alcohol than the youngest age group (baseline = aged 20-35 years), although the differences are not significant for the 45-55 and the 55-65 age groups. The oldest cohorts are less likely to consume alcohol than the youngest after controlling for age and other variables with increasingly negative effects toward younger cohorts. Income has a positive effect on alcohol participation. Women whose parents are farmers, whose mothers are working class or self-employed, and those who did not report the status of the mother, are less likely to participate in alcohol consumption than those with white-collar parents. Women who did not report their father's socioeconomic status are more likely to participate in alcohol consumption. The more children a woman has, the less likely she is to participate in alcohol consumption. Women with moderate health in W1 are less likely to consume alcohol than those with good health. Finally, there is a tendency to a decrease in participation in alcohol consumption over time according to the wave dummy.

**Table 5:** Results of the decomposition analysis

Women		W2					W3				
**Variables**:	coefficient	elasticity	(C)	contrib	%contrib		elasticity	(C)	contrib	%contrib	
**Age**	**(2)**	**(3)**	**(4)**	**(5)**	**(6)**	**(7)**	**(3)**	**(4)**	**(5)**	**(6)**	**(7)**
35< = age < 45	0.913**	0.2809	-0.0247	-0.0069	-2.00	10.37	0.2364	-0.1993	-0.0471	-15.77	2.72
45< = age < 55	0.747	0.1725	0.1417	0.0244	7.06		0.2541	0.0276	0.0070	2.35	
55< = age < 65	0.878	0.1975	0.0280	0.0055	1.60		0.2039	0.1384	0.0282	9.45	
65< = age < 75	1.661*	0.4009	0.0068	0.0027	0.79		0.3761	0.0038	0.0014	0.48	
75< = age < = 84	2.021*	0.1157	0.0875	0.0101	2.93		0.4081	0.0454	0.0185	6.21	
**Cohort**											
1932< = cohort < 1947	-1.389***	-0.5141	0.1140	-0.0586	-16.94	-25.15	-0.5474	0.1177	-0.0645	-21.59	-30.69
1924< = cohort < 1932	-1.764***	-0.3125	0.0431	-0.0135	-3.89		-0.3155	0.0318	-0.0100	-3.36	
1916< = cohort < 1924	-2.986***	-0.5887	-0.0218	0.0128	3.70		-0.5067	-0.0078	0.0039	1.32	
Cohort < 1916	-3.926***	-0.3616	0.0768	-0.0278	-8.02		-0.2671	0.0790	-0.0211	-7.07	
**Father's socioeconomic status**											
Working class	-0.338	-0.1818	-0.0722	0.0131	3.79	10.90	-0.1861	-0.0763	0.0142	4.75	12.40
Self-employed	0.275	0.0503	0.0541	0.0027	0.79		0.0517	0.0383	0.0020	0.66	
Farmer	-1.052***	-0.2440	-0.0552	0.0135	3.89		-0.2426	-0.0506	0.0123	4.11	
Missing	1.982***	0.1263	0.0665	0.0084	2.43		0.0875	0.0978	0.0086	2.87	
**Mother's socioeconomic status**											
Working class	-1.145**	-0.2589	-0.0856	0.0221	6.40	3.36	-0.2714	-0.0685	0.0186	6.23	3.29
Self-employed	-1.394*	-0.0683	0.0157	-0.0011	-0.31		-0.0675	0.0414	-0.0028	-0.93	
Farmer	-2.060***	-0.1824	-0.1149	0.0210	6.06		-0.1835	-0.0852	0.0156	5.24	
Missing	-1.212**	-0.9487	0.0321	-0.0304	-8.79		-0.9383	0.0230	-0.0216	-7.24	
**Immigrant**											
First-generation immigrant	-0.0348	-0.0033	-0.0148	0.0000	0.01	0.01	-0.0032	-0.0131	0.0000	0.01	0.01
**Marital status**											
Single	-0.151	-0.0515	0.3996	-0.0206	-5.94	-5.94	-0.0606	0.3309	-0.0201	-6.72	-6.72
**Children**											
One child	-0.703*	-0.1271	0.0143	-0.0018	-0.53	23.39	-0.0853	-0.1923	0.0164	5.50	27.03
Two children	-0.935*	-0.1876	-0.1292	0.0242	7.00		-0.1075	-0.2868	0.0308	10.32	
Three or more children	-1.544**	-0.1522	-0.3844	0.0585	16.91		-0.0650	-0.5155	0.0335	11.21	
**Education**											
Vocational high school studies	0.229	0.0955	-0.0530	-0.0051	-1.46	0.30	0.1002	-0.0716	-0.0072	-2.40	0.59
Academic high school level	-0.405	-0.0280	0.0860	-0.0024	-0.70		-0.0264	-0.0036	0.0001	0.03	
University level	0.127	0.0349	0.2438	0.0085	2.46		0.0388	0.2278	0.0088	2.96	
**Income**											
Ln of full income	0.0413	0.5831	0.0173	0.0101	2.92	100.23	0.5929	0.0232	0.0138	4.61	119.65
Mean of Ln of full income	1.190***	16.8573	0.0200	0.3367	97.32		17.0071	0.0202	0.3435	115.03	
**Occupation**											
Self-employed	-0.282	-0.0117	-0.2625	0.0031	0.88	2.14	-0.0010	-0.5860	0.0006	0.19	-4.52
Student	2.309*	0.0218	0.0351	0.0008	0.22		0.0243	-0.4030	-0.0098	-3.28	
Unemployed	0.655	0.0120	-0.1475	-0.0018	-0.51		0.0276	-0.1817	-0.0050	-1.68	
Retired	-0.219	-0.0784	-0.0043	0.0003	0.10		-0.1107	0.0133	-0.0015	-0.49	
Homemaker	-0.147	-0.0096	-0.5213	0.0050	1.45		-0.0101	-0.2177	0.0022	0.73	
**Period**											
Wave dummy	-0.759***	0.0000	.	.	0.00	0.00	-0.9557	0.0000	0.0000	0.00	0.00
**Health**											
Moderate health	-0.591**	-0.1386	-0.0349	0.0048	1.40	2.11	-0.1269	-0.0523	0.0066	2.22	2.58
Bad health	-0.590	-0.0296	-0.0831	0.0025	0.71		-0.0240	-0.0444	0.0011	0.36	
**Individual heterogeneity**											
IH	P* = 0.0569					-21.71					-26.33
Total				0.3460	100.00	100.00			0.2986	100.00	100.00

*There is no parameter for the individual heterogeneity, by definition; consequently we report the p-value instead.

### Decomposition analysis of alcohol participation concentration indices

Following equation 4, the Cs for each wave are decomposed into contributions from the explanatory variables from the random effects Mundlak model. The results are reported in Table 5, columns 3-7; it shows the Cs and elasticities for each determinant, in both absolute and percentage contributions.

If the value of the contribution of variable X is x and positive (negative), then the inequality in alcohol participation would decrease (increase) by x% if the variable was to become equally distributed across the income distribution (C equal to 0) or the elasticity of the variable was to equal zero (parameter of the regression is equal to 0). The row total at the bottom of Table 5 shows the explained C, which is the contribution of the explanatory variables to C; the explained C is positive, decreasing over time (like the "true" C), and larger than the "true" C. Income and having children contribute to a pro-rich inequality, while IH contributes to pro-poor inequality. Education, however, does not have a large contribution to the inequality. Father's and mother's socioeconomic group appear to positively influence participation in alcohol consumption.

### Total differential approach (TDA)

This section presents the TDA results. Column 8 in Table 6 shows the aggregated percentage of a variable's contribution to C. If it is negative (positive), it means that the variable contributes to decreases (increases) in the Cs. Age contributes to a decrease in C, while income contributes to an increase in C. Occupation has a negative impact in the participation model. Columns 1 and 3 correspond to the effect of a change in the mean and a change in the dispersion of the characteristic along the distribution of income respectively.

**Table 6:** Changes in inequalities following the total differential approach

Variables	M effect	%	C effect	%	Residual	dtotal	%	Agg. %
	(1)	(2)	(3)	(4)	(5)	(6)	(7)	(8)
**Age**								-125.74
35< = age < 45	0.0081	17.02	-0.0490	-103.42		-0.0410	-86.40	
45< = age < 55	-0.0005	-1.13	-0.0197	-41.49		-0.0202	-42.62	
55< = age < 65	-0.0005	-1.16	0.0218	46.00		0.0213	44.84	
65< = age < 75	0.0040	8.38	-0.0012	-2,.4		0.0028	5.84	
75< = age < = 84	-0.0176	-37.12	-0.0049	-10.27		-0.0225	-47.40	
**Cohort**								-59.17
1932< = cohort < 1947	0.0010	2.07	-0.0019	-4.04		-0.0009	-1.97	
1924< = cohort < 1932	0.0000	0.07	0.0035	7.40		0.0035	7.47	
1916< = cohort < 1924	-0.0147	-31.01	-0.0082	-17.33		-0.0229	-48.35	
Cohort < 1916	-0.0069	-14.64	-0.0008	-1.69		-0.0077	-16.33	
**Father's socioeconomic status**								12.32
Working class	0.0006	1.23	0.0007	1.57		0.0013	2.80	
Self-employed	-0.0001	-0.18	-0.0008	-1.67		-0.0009	-1.86	
Farmer	-0.0007	-1.48	-0.0011	-2.33		-0.0018	-3.82	
Missing	0.0032	6.84	0.0040	8.36		0.0072	15.19	
**Mother's socioeconomic status**								-6.60
Working class	0.0024	5.02	-0.0044	-9.31		-0.0020	-4.30	
Self-employed	-0.0002	-0.40	-0.0018	-3.70		-0.0019	-4.10	
Farmer	-0.0001	-0.30	-0.0054	-11.42		-0.0056	-11.71	
Missing	-0.0022	-4.55	0.0086	18.06		0.0064	13.51	
**Immigrant**								-0.04
First-generation immigrant	-0.0000	-0.03	-0.0000	-0.01		-0.0000	-0.04	
**Marital status**								2.90
Single	-0.0022	-4.56	0.0035	7.46		0.0014	2.90	
**Children**								1.64
One child	-0.0057	-12.01	0.0263	55.38		0.0206	43.37	
Two children	-0.0225	-47.45	0.0296	62.33		0.0071	14.88	
Three or more children	-0.0468	-98.67	0.0199	42.06		-0.0268	-56.61	
**Education**								-0.81
Vocational high school studies	-0.0008	-1.65	-0.0018	-3.74		-0.0026	-5.39	
Academic high school level	-0.0001	-0.24	0.0025	5.29		0.0024	5.05	
University level	0.0003	0.71	-0.0006	-1.18		-0.0002	-0.47	
**Income**								13.38
Ln of full income	-0.0006	-1.27	0.0035	7.31		0.0029	6.04	
Mean of Ln of full income	-0.0003	-0.53	0.0037	7.87		0.0035	7.34	
**Occupation**								-30.90
Self-employed	-0.0044	-9.26	0.0038	7.96		-0.0006	-1.30	
Student	-0.0003	-0.54	-0.0096	-20.15		-0.0098	-20.69	
Unemployed	-0.0045	-9.58	-0.0004	-0.86		-0.0050	-10.44	
Retired	0.0048	10.08	-0.0014	-2.92		0.0034	7.16	
Homemaker	0.0003	0.54	-0.0029	-6.16		-0.0027	-5.62	
**Period**								0.00
Wave dummy	0.0000	0.00	0.0000	0.00		0.0000	0.00	
**Health**								-5.14
Moderate health	-0.0023	-4.95	0.0024	5.06		0.0001	0.11	
Bad health	-0.0013	-2.84	-0.0011	-2.42		-0.0025	-5.26	
								
**IH**	-0,0119	-25.03	-0.0081	-17.14		-0.0200	-42.17	-42.17
Total column	-0.1226		0.0087		0.0665	-0.1140	-240.34	-240.34
Difference in C over waves						**-0.0474**		
% Total	-258.63	-258.63	18.29	18.29	140.3383		-100.00	

We take the age effect on participation in alcohol consumption as an example. Age is the largest contributor (-125.7%) to the decrease in participation inequality. If we had observed no changes in distribution of people aged 35-45 over income between W2 and W3, the income-related inequality in alcohol consumption would have increased by 0.0490. This represents 103% of the total change (columns 6 and 7, first row), which is due to the change of this age group in the income distribution. We can say that this change in the distribution of this age group over income between waves contributes to a decrease in alcohol-related inequality.

The row showing difference in C over waves at the bottom of Table 6 is the difference in the explained C (-0.0474), which is 0.3460 for W2 and 0.2986 for W3. The total value of column 6 (-0.1140) gives the approximate change in C explained by the TDA. The difference between the change explained by the TDA and the change of the explained C represents 140% of the change in the explained C. In a nutshell, most of the changes are due to changes in means; means contribute to a decrease of 259% which is counteracted by residuals (+140%) and by the Cs of the independent variables (+18%).

A large part of the decrease in the inequality of participation in alcohol consumption is due to age and cohort. Within those two variables, the contributors to changes are the increase in C among the 35-45 age group (from -0.02 to -0.2), the increase in the proportion of older people, and the decrease of the older cohorts. Another part is due to IH (-42.2%); which by definition is not explained. For students, observing no differences in C would have led to an increase by 0.0098 (20%). Within the variables for children, inequalities of women with one child contribute to an increase of 0.0263 in the alcohol-related inequality. This effect is neutralised by the direct effect of the mean for women with more than three children. Change in inequalities within income contributes to an increase in participation inequality (7.87%).

### Sensitivity analyses

Our interpretations of the Oaxaca decomposition are similar to the TDA results. The advantage of the TDA is that we can distinguish between the effect of the mean and the concentration index of the independent variable. The results of the TDA (see above) are more informative than the Oaxaca decomposition, which in this case concludes that most of the inequality is explained by the elasticity.

### Male results

On one hand, there are some similarities between men and women; for example, income is also the larger contributor for men, while on the other hand there are some dissimilarities; for example, education is a large contributor in the male model but not in the female model (higher education contributes to a pro-poor inequality). This shows at the very least that determinants among men and women are different, meaning that the results for men should not be deduced from the analyses of women (detailed results of the male analyses are available from the authors upon request).

### Intensity model

Our dataset does include some information on the level of alcohol consumption, but the questions used to measure this intensity differ between the waves. Consequently, the results should be interpreted with caution. The C is positive, and decreases over time, though the decrease is not significant. Income is the largest contributor to the concentration index. Age contributes to a decrease in C over time, while income contributes to an increase of the inequality.

### Attrition analysis

We performed a regression on the W2 observations, with the dependent variable being dropout in the next wave and the independent variables being the same as those for the main regression, including participation in alcohol consumption. The individuals who disappear in W3 are likely to be older, in poorer health, unemployed, retired, and without a university education. Participating in alcohol consumption lowers the probability of being a dropout.

## Discussion

The main result of the study is that inequality in alcohol participation has a pro-rich inequality; that is, participation in alcohol consumption is more concentrated among the rich. This result is consistent with some of the articles cited in the background section. The characteristics explaining this inequality at different time points remain the same over the waves, mainly income and having children, while IH contributes to pro-poor inequality.

The inequality decreases over time when computed using a measure of long-term income, but increases over time when computed on the basis of short-term current income. However, this change is not statistically significant. The reason for the differences in pattern over time is the effect of alcohol-related income mobility on short-term income measures. Concerning our results, it means that there were more alcohol consumers who moved to higher income groups than there were non-consumers who moved to lower income groups. Since we were not particularly interested in this effect in the present study, our analyses are mainly based on the long-term income measure, resulting in a decrease in the pro-rich inequality of alcohol participation.

There are two main results from the TDA. The first is that most of the change is due to changes in means, and all means apart from income are proportions; consequently, the changes in the participation C are due to changes in the relative importance of population groups (e.g. decrease in proportion of the eldest cohorts, or of women with three or more children) and not to changes in their distribution by income. The second result is that unexplained factors (IH and the residual effect) are large, showing that there are some uncontrolled characteristics of the population that contribute to changes.

In terms of known characteristics, the two major contributors to a change in inequality are age and cohort. Within those two contributors, the strongest characteristics leading to a decrease of the inequality include the increase in the proportion of older people and the decrease in proportion of the oldest cohorts. Chandola *et al. *[42] found an increase in social inequalities in perceived health between middle age and early old age. The decrease in income-related alcohol inequalities that we found should also lead to an increase in health inequality (see paragraph below); and it is driven by an ageing population (decrease in the proportion of the oldest cohorts and increase in the proportion of older people). Our results also show that the increase in inequality among the 35-45 age group is another contributor to a decrease of income-related alcohol inequality.

It is not obvious whether participation in alcohol consumption is beneficial or detrimental to health. The pattern and level of consumption are important, among other variables, as are the characteristics of the consumer. We expect, for example, that participation is more likely to be beneficial in older age groups and more likely to be detrimental in younger age groups [8,43]. Although this aspect is not important for the current study as such, it must be considered when the results are discussed in relation to the inequalities in health. If we assume that the net effect of alcohol consumption, compared to abstention, is detrimental, an assumption that is not controversial if based on the epidemiological literature, then the pro-rich inequality in alcohol participation should reduce the health inequality in Swedish society. The fact that the inequality in alcohol participation has decreased over the study period, mainly due to changes in the proportions of population groups rather than changes in wealth patterns, is thus expected to play a role in the coinciding increase in health inequalities. Inequality in alcohol participation is thus an important determinant of health inequalities, although maybe not in the direction one would assume a priori. A consequence of this would be that, from a policy perspective where reduced inequalities in health are of primary importance, the reduction in alcohol participation inequality is unwanted. It is thus important to not only look at inequality measures in themselves but also to examine how they affect other outcomes as determinants. A discussion we will only mention here is whether a hypothetical intervention resulting in Pareto improvements reducing inequalities in alcohol participation should be implemented even though it would increase inequalities in health. Economists are generally in favour of Pareto improvements for obvious reasons, although the policy/political decision might be much more complicated.

One limitation of the study connected to the alcohol variables is that we have not been able to control for reversed causality. Most problematic is that current abstainers can be former heavy drinkers, and thus suffer from the adverse effects from drinking while being categorised as abstainers. Jarl & Gerdtham [44], who examined the same waves in the same data set showed that, very basically, no individuals moved from heavy consumption (>20gr pure alcohol per day for women and >40 for men) to abstinence between waves 2 and 3, while about 5% moved from low consumption to abstinence. It should be noted that the current study does not use exactly the same sample as Jarl & Gerdtham [44], and that it is possible that former heavy drinkers who reduced their consumption before wave 2 are coded as abstainers, but it does still suggest that the former drinker error is of lesser concern in the context of the current study. However, if the present study is in fact biased due to the former drinker error, this could potentially explain why, with the assumption that heavy alcohol consumption reduces human capital, alcohol participation is more concentrated among the rich. Future studies must therefore endeavour to control for reversed causality.

Finally, the significance of the IH is assessed by the mean of independent variables, though it is only significant at the 0.10 level. However, a FE specification is not comparable to RE, as it only takes into account people with at least two observations. Consequently, as there is some dropout between the two waves (17.9%), the RE technique uses more information than the FE. The mean of Ln of income could have been included in IH, but we would have lost another interpretation that can be relevant concerning mean of income: that as a permanent income. As a consequence, the study shows that permanent income is more significant and contributes more to the income-related inequality in alcohol consumption than current income.

The effect of inequalities in alcohol consumption on the inequalities in health should be interpreted with caution, even with the assumption of a detrimental effect of consumption. For example, the harming effect of the same quantity might be different between population subgroups. A study by Bloomfield et al. [45], and a commentary by Mäkelä [43] emphasise that the more disadvantaged subgroups of the population suffer from higher levels of alcohol-related harm. Moreover, a fat-rich diet has been shown to be a risk factor in the development of alcoholic liver disease [46]. Given that diet differs between rich and poor, the increased harm connected to being poor could be due to this interaction. On top of this, we do not have a consistent measure on how people consume alcohol (frequency), we do not have a consistent measure on the type of alcohol they consume (a glass of wine may not have the same effect as a pint of beer), and we do not have a reliable quantity variable. All this should be taken as cause for caution, indicating the complexity of alcohol consumption and its effects. It would be of interest to study alcohol-related harm inequality, as it could give information about differences in harmful effects of alcohol between population groups, which better corresponds to health inequalities. This is, however, left to future research.

## Conclusions

This study shows that there is a decreasing pro-rich inequality in alcohol participation, and that this decrease is mainly due to changes in proportions of population groups. As the pro-rich inequality is expected to reduce health inequalities, it follows that interventions that are mostly effective in reducing alcohol consumption among high-income population group would increase health inequalities. Research on the subject using a quantitative variable for harmful consumption would be of much interest. At the moment, there is insufficient evidence to give policy makers an indication of the effect on health inequalities of reducing alcohol consumption. However, alcohol has been shown elsewhere to be harmful on its own, independently of income levels. The inequality in participation might therefore be considered to actually reduce overall health inequalities.

## List of Abbreviations

TDA: Total Differential Approach; C: Concentration Index; IH: Individual Heterogeneity; RE: Random Effect; FE: Fixed Effect; GLS: Generalised Least Square; OECD: Organisation for Economic Co-operation and Development; W1: Wave 1; W2: Wave 2; W3: Wave 3

## Authors' contributions

All authors took an equal part in designing the study. JBC carried out the statistical analysis and wrote the draft manuscript. UGG revised the manuscript and made significant improvements regarding the methods, introduction, and discussion. JJ revised the manuscript and made significant improvements regarding the introduction and discussion. All authors have approved the final version.
